# Sibling-controlled study of the impact of dietary therapy on the gut microbiota in children with phenylketonuria

**DOI:** 10.3389/fnut.2025.1662634

**Published:** 2026-01-16

**Authors:** Natsuki Ohmi-Shimizu, Chika Takano, Noriko M. Tsuji, Motoko Iwama, Naoko Okamura, Shihoko Komine-Aizawa, Satoshi Hayakawa, Erika Ogawa, Ichiro Morioka, Mika Ishige

**Affiliations:** 1 Department of Pediatrics and Child Health, Nihon University School of Medicine, Tokyo, Japan; 2 Division of Microbiology, Department of Pathology and Microbiology, Nihon University School of Medicine, Tokyo, Japan; 3 Division of Immune Homeostasis, Department of Pathology and Microbiology, Nihon University School of Medicine, Tokyo, Japan; 4 Department of Food Science, Jumonji University, Saitama, Japan; 5 Division of Nutritional Management, Nihon University Hospital, Tokyo, Japan; 6 University Research Center, Nihon University, Tokyo, Japan; 7 Department of Pediatrics, Tokyo Metropolitan Hiroo Hospital, Tokyo, Japan

**Keywords:** phenylketonuria, diet therapy, gut microbiota, food allergy, short-chain fatty acids

## Abstract

**Background:**

Phenylketonuria (PKU) is an autosomal recessive metabolic disorder caused by a deficiency of phenylalanine hydroxylase activity. Due to intolerance to the dietary intake of phenylalanine (Phe), the patients need to take a low-protein diet alongside immediate utilization of Phe-free medical formula upon diagnosis to maintain optimal plasma Phe concentrations. While dietary influences on gut microbiome composition are well-established, the potential alterations in microbiota and their impact on the immune function of children with PKU remain underexplored. We therefore conducted a pilot, sibling-controlled study to assess how dietary therapy for PKU affects gut microbiota and whether these changes are associated with food allergy incidence.

**Materials and methods:**

A questionnaire-based survey was conducted across multiple institutions to determine the prevalence of food allergies in children with PKU. Four children with PKU who have unaffected siblings were recruited to investigate their dietary intake and immunological profiles. Stool samples from both groups were collected and analyzed for gut microbiota composition and short-chain fatty acid (SCFA) profiles.

**Results:**

The survey indicated a notably low prevalence of food allergies in children with PKU (approximately 1%). The four children with PKU strictly adhered to a low-protein diet and maintained their blood phenylalanine levels within the target therapeutic range. Among the PKU group, only one child had an egg allergy, while the remaining children showed no allergic tendencies. Although no adverse immunological effects were observed, the gut microbiota composition of the PKU group significantly differed from that of the unaffected siblings, as indicated by the weighted UniFrac distance (*p* = 0.027). In the PKU group, the abundance of *Faecalibacterium prausnitzii* was significantly reduced (*p* = 0.002), that of *Bifidobacterium* was increased, and *Akkermansia muciniphila* was detected. No overall decrease in total SCFA levels was observed in the PKU group, although the acetate/butyrate ratio significantly increased.

**Discussion:**

This study is the first to characterize the gut microbiota of children with PKU using their unaffected siblings as genetically and environmentally matched controls. Our findings suggest that the distinctive dietary management in PKU results in a characteristic gut microbial profile. We further propose a novel hypothesis that these compositional shifts may establish a unique intestinal microenvironment in diet-adherent PKU, which could be negatively associated with the development of food allergy. Larger cohort studies incorporating host metabolomic profiling are needed to determine causal links between dietary therapy and immunological background, ultimately contributing to improved nutritional management.

## 1 Introduction

Phenylketonuria (PKU; OMIM 261600) is an inherited metabolic disorder caused by a mutation in phenylalanine hydroxylase enzyme (PAH), which converts phenylalanine (Phe) into tyrosine. Since Phe is not metabolized in PKU patients, phenylalanine accumulates in the blood and is toxic to the brain (1). Untreated PKU leads to neurodevelopmental damage and behavioral problems; hence, diagnosis and treatment should be initiated as early as possible (2). Dietary therapy is the primary treatment for PKU. Due to their inability to metabolize dietary Phe, patients with PKU must adhere to a low-protein diet and immediately use Phe-free medical formulas upon diagnosis to maintain optimal plasma Phe concentrations.

Our institution has been involved in the dietary management of over 100 patients with PKU. Recently, we encountered a child with both PKU and food allergy, which was the first case in our experience. In contrast, the prevalence of food allergies has been increasing in Japan in recent years. Although various factors, including environmental or genetic factors, are involved in the onset of food allergies, it was previously considered that immaturity of the digestive function and other factors influenced the establishment of allergic sensitization. However, the recent hypothesis suggests that sensitization to allergens occurs through environmental exposure to allergens through the skin and that consumption of food allergens induces oral tolerance (3). This hypothesis is increasingly evident with supportive observations like the learning early about peanut allergy (LEAP) (4) and Tow-step egg introduction for prevention of egg allergy in high-risk infants with eczema (PETIT) studies (5). In children with PKU, strict nutritional management often delays the introduction of natural foods, which could theoretically influence the risk of food allergy development. Contrary to this expectation, however, our clinical experience indicates that food allergy is extremely rare in PKU patients. To our knowledge, no studies have directly examined the relationship between PKU and food allergy, and there are no reports of food allergy developing after liberalization of natural protein intake with sapropterin or pegvaliase. Similarly, no such cases have been observed in our cohort. Notably, even in clinical trials of pegvaliase (6), which included detailed adverse event monitoring, food allergy was not reported. These observations raised the idea that PKU dietary management may serve as a protective factor against the onset of food allergies.

If children with PKU show a particular tendency towards food allergies, the underlying mechanism may be related to the gut microbiota, as diet alters the composition of the gut microbiota. Byproducts of microbial metabolism can interact with the host metabolism to change gut function locally and distally by acting on the liver, brain, muscle, and adipose tissues (7). Alterations in gut microbial composition and diversity, referred to as dysbiosis, have been implicated in the pathogenesis of various diseases, including obesity, allergic disorders, and inflammatory bowel disease. To date, only a limited number of studies have applied 16S rRNA sequencing to characterize the gut microbial communities of individuals with PKU. Pinheiro de Oliveira et al. studied a small cohort (8 patients with PKU and 10 healthy controls), although interpretation was limited by confounders such as antibiotic use and inclusion of breastfeeding children (8). Bassanini et al. analyzed a larger cohort (21 patients with PKU and 21 with mild hyperphenylalaninemia [HPA]) and compared them with and without diet therapy (9). They demonstrated shifts in Firmicutes populations and suggested that both the quality and quantity of carbohydrate intake contribute to microbiome changes (8, 9). Mancilla et al. conducted the first study of adults with PKU compared with non-PKU controls, reporting characteristic microbial shifts with relative enrichment of *Bifidobacterium*, *Bacillus*, *Alistipes*, *Clostridium*, *Akkermansia*, and *Bacteroides*, and depletion of *Lactobacillus*, *Porphyromonas*, *Frisingicoccus*, *Blautia*, and *Faecalibacterium* (10). A subsequent cross-sectional study comparing adults with PKU on a traditional Phe-restricted diet with those treated with pegvaliase (Palynziq^®^) on a liberalized diet demonstrated marked differences in gut microbiome composition, indicating the role of dietary therapy in shaping the gut microbiota in PKU (11). Because diets vary depending on the region and cultural background in which they reside, the changes in gut microbiota in Japanese patients with PKU with diets different from those reported in previous studies remain poorly understood. Furthermore, the comparison between PKU and HPA may still be subject to bias due to environmental and genetic backgrounds. Therefore, we considered that unaffected siblings of patients with PKU should be enrolled as the healthy control to minimize their maternal and ecological biases.

The primary aim of this study was to evaluate the impact of dietary therapy for PKU on the gut microbiota using an appropriate control group. The secondary aim was to discuss the possibility that alterations in the gut microbiota of children with PKU might be associated with food allergy. We first conducted a questionnaire survey on food allergies at several institutions to investigate the prevalence of food allergies in PKU. We then enrolled four children with PKU who have unaffected siblings and investigated their diet and immunological profiles. Finally, we analyzed and compared the gut microbiota and short-chain fatty acid (SCFA) levels in children with PKU and their unaffected siblings.

## 2 Materials and methods

### 2.1 Questionnaire design

A questionnaire was administered to investigate the association between PKU and allergic diseases. Specifically, we surveyed physicians at hospitals that treat a large number of patients with congenital metabolic disorders throughout Japan. The questionnaire included the following: (a) number of PKU patients; (b-1) number of children with food allergies in (a); and (b-2) foods that cause food allergies. Food allergy was defined as a physician-diagnosed, immunologically mediated reaction to a specific food with clinical symptoms; asymptomatic sensitization was not counted as food allergy in this study.

### 2.2 Study design and participant selection

This study was conducted in the Department of Pediatrics, Nihon University Hospital, Tokyo, Japan. Patients with classical PKU aged 3–7 years who met the following criteria were recruited: (a) adherence to dietary therapy since birth following diagnosis, (b) not breastfeeding and consuming three meals a day, (c) strict adherence to diet therapy and maintenance of plasma Phe levels within the therapeutic target range, and (d) had an unaffected sibling within 3 years of each other. Finally, eight children (four pairs of individuals with PKU and their unaffected siblings) were enrolled in the study. Four patients with PKU were included in the PKU group, and four unaffected siblings from each group were included in the Sib group. The study protocol was approved by the Ethics Committee of Nihon University School of Medicine (No. 2021-08-02). Informed consent was obtained from all patients and their unaffected siblings and/or legal guardians. Clinical information, such as age and birth history, and clinical parameters, such as plasma Phe levels and eosinophil count, were obtained by reviewing medical records. The plasma Phe levels and eosinophil counts were calculated as the average of five measurements. Three-day dietary records were filled out by a parent for each enrolled subject, and the average amounts of energy and nutrient intake (carbohydrate, protein, and fat) were calculated by our institute’s dietician.

### 2.3 Immunological profiles

Blood samples were collected from children with PKU, and their non-specific IgE levels were measured during periodic hospital inspections. Serum-specific IgE levels were analyzed using a MAST 48 mix. Blood samples were collected in heparinized tubes to determine peripheral blood lymphocyte subsets. Lymphocytes were stained with monoclonal antibodies against CD3, CD4, CD8, CD19, and CD25, and intracellular staining for FoxP3 was performed. Peripheral blood lymphocyte subsets and serum-specific IgE levels were analyzed by SRL, Inc.

### 2.4 Gut microbiome analysis

Fecal samples were collected twice from children with PKU and their siblings. Samples were collected at home and immediately placed into a preservative solution containing guanidine thiocyanate and detergent (Metabolokeeper^®^; Techno Suruga Laboratory Co., Ltd., Shizuoka, Japan). Samples were stored at 4 °C until DNA extraction. To assess reproducibility, the two fecal collections were performed more than one year apart. The sample collection succeeded in three families and once in one family. Gut microbiome analysis was performed by TechnoSuruga Laboratory Co., Ltd. Fecal DNA was extracted as described by Takahashi et al. (12). DNA samples were prepared using a NanoDrop ND-8000 spectrophotometer (Thermo Fisher Scientific). Next, the V3–V4 hypervariable regions of the bacterial 16S rRNA gene were amplified using the primers Pro341F-Pro805R (12) and dual-index (8-bp barcode) (13). The PCR amplification conditions were in accordance with the method proposed by Takahashi et al. (12). The sequences of the amplified DNA were determined using the MiSeq platform (Illumina, USA) and the sequencing kit of an Illumina MiSeq Reagent Kit v3 (600 cycles) for approximately 430 bp, excluding primers. The primer sequences on paired-end sequencing reads were trimmed by cutadapt version 1.18 with default settings (14). Paired-end sequencing reads were merged using fastq-join version 1.3.1 program with default settings (15). The joined amplicon sequence reads were processed through QIIME2 version 2020.6 (16). The quality filtering and deletion of chimeric sequences were done, and then representative sequences were created using DADA2 (Divisive Amplicon Denoising Algorithm 2) denoise-single version 1.10.0 with default settings (17). Taxonomy of representative sequences was assigned using Greengenes Database version 13.8 (18) by training a Naive Bayes classifier. The samples were rarefied to a minimum of 13,053 sequences per sample, and alpha diversity indices (Chao1, Shannon, and Simpson) and beta diversity metric (weighted UniFrac, unweighted UniFrac, and Bray–Curtis distances) were calculated. The statistical significance of Chao1, Shannon, and Simpson indices among groups was assessed by Kruskal–Wallis test. The statistical significance of similarity of bacterial communities among groups was assessed with ANOSIM test. The *p*-values obtained from statistical tests were adjusted for multiple comparisons using the Benjamini–Hochberg false discovery rate (FDR) method, and the resulting adjusted values are reported as q-value.

### 2.5 Fecal metabolite measurement

Fecal samples were collected from three children with PKU and their unaffected siblings. SCFA levels were analyzed by TechnoSuruga Laboratory Co., Ltd. The concentration of each SCFA, including acetic, propionic, isobutyric, butyric, isovaleric, and valeric acid, was assessed using high-performance liquid chromatography. A fixed amount of specimen was weighed in bead tubes, suspended in the extraction solution, and heated at 85 °C for 15 min. After crushing with beads, the sample solution was centrifuged (18,400 × g, 10 min), and the supernatant was filtered through a 0.20-μm membrane filter. The sample solutions were analyzed using a Shimadzu organic acid analysis system (Shimadzu, Japan) and an electrical conductivity detector (CDD-10Avp). The results were expressed as mg/g wet weight of feces. The lower limit of quantification was set at 0.05 mg/g for acetic acid and propionic acid and 0.1 mg/g for isobutyric acid, butyric acid, isovaleric acid, and valeric acid.

### 2.6 Statistical analysis

Statistical analyses were performed using JMP software (version 18.2.2) and R (version 4.1.2). For diversity indices and comparisons of bacterial taxa (n ≥ 7 per group), data were analyzed using the Mann–Whitney U test, and *p*-values < 0.05 were considered statistically significant. For SCFA levels, sample sizes were extremely small (n = 3 per group); therefore, hypothesis testing was considered exploratory. For each metabolite, the observed difference in medians between the two groups was calculated, and a bootstrap procedure with 10,000 resamples was applied. Two-sided *p*-values were estimated as the proportion of bootstrap replicates with an absolute difference greater than or equal to the observed difference, and 95% confidence intervals were derived using the percentile method.

## 3 Results

### 3.1 Questionnaire results

The results of the allergy surveys conducted at six facilities, including our hospital and five other hospitals, are shown in Table 1. Of the approximately 90 children with PKU reported, only one had a food allergy. Thus, the prevalence of food allergies in children with PKU was approximately 1%. This patient was enrolled in this study as PKU-4 and had an allergic symptom of eczema around the mouth immediately after ingesting food containing eggs, but had already gone into remission at the time of this study.

**Table 1:** Results of the questionnaire on food allergy in patients with PKU.

Medical institution	Number of patients with PKU	Number of patients affected by food allergy
Our hospital	25	1 (egg)
A	11	0
B	2	0
C	18	0
D	30	0
E	6	0
Total	92	1

### 3.2 Patient background and nutritional assessment

The cohort characteristics of eight participants are shown in Table 2. The body mass index was normal in the PKU group. All subjects with PKU maintained plasma Phe levels within the therapeutic target range (120–360 μmol/L). The average Phe levels of PKU 1–4 among the five visits were 179.1 μmol/L, 43.6 μmol/L, 164.9 μmol/L, and 166.9 μmol/L, respectively. Table 3 shows the nutritional assessment results of patients with PKU. The amounts of protein and carbohydrates in the Phe-free formula and natural food were calculated from the PKU three-day dietary records. The natural protein intake of patients with PKU was limited to 2.0 g/day or 4.0 g/day, depending on their age. By consuming the Phe-free formula, patients with PKU achieved energy intake consistent with age-appropriate recommendations. Detailed three-day food records for siblings were not consistently available; however, where collected, nutrient intakes generally met recommended levels. For reference, the Dietary Reference Intakes for Japan (2020) issued by the Ministry of Health, Labor, and Welfare are provided in Table 4. As expected, natural food intake differed significantly between the PKU and Sib groups. Children with PKU consumed substantially less natural protein and a relatively higher proportion of carbohydrate. In healthy children, the dietary protein-to-carbohydrate ratio is approximately 1:3.5, whereas in children with PKU, the ratio was approximately 1:30, reflecting the restriction of protein from natural food sources.

**Table 2:** Patient background.

	Family no.	1	2	3	4
Age (y)	PKU	3	6	7	7
	Sib	5	8	7	8
Height (SD) body mass index	PKU	−0.51 SD Kaup index 16.1	−0.96 SD Rohrer index 150	−1.47 SD Rohrer index 150	−0.59 SD Rohrer index 146
	Sib	–	–	–	–
Birth history	PKU	Vaginal delivery	Vaginal delivery	C-section	Vaginal delivery
	Sib	Vaginal delivery	Vaginal delivery	C-section	Vaginal delivery

**Table 3:** Nutritional assessment of patients with PKU.

PKU patient no.		1	2	3	4
Age (y)		3	6	7	7
Phe-free formula	Phe-free protein (g/day)	25.3	32.8	32.1	33.7
	Phe-free carbohydrate (g/day)	96.7	96.7	90.6	96.7
Natural food	Protein (g/day)	1.9	4.1	5.9	5.7
	Carbohydrate (g/day)	64.3	158.5	157.6	122
	Protein: Carbohydrate ratio	1:34	1:39	1:27	1:21
	PFC* ratio (%)	2:15:83	2:21:77	3:17:80	4:20:76
Total	PFC ratio (%)	10:31:59	9:26:64	10:24:66	10:26:64

*PFC ratio = percent of total energy from Protein: Fat: Carbohydrate.

**Table 4:** Recommended dietary allowance for healthy children in Japan.

Age	Protein (g/day)	Protein (% energy)	Fat (% energy)	Carbohydrate (% energy)	Protein: carbohydrate ratio
3 ~ 5	25	13 ~ 20	20 ~ 30	50 ~ 65	1: 3.5
6 ~ 7	30	13 ~ 20	20 ~ 30	50 ~ 65	

### 3.3 Immunological profiles

As shown in Table 5, the levels of eosinophils and non-specific IgE were normal in all three patients. Patient PKU-4 showed a relatively high number of eosinophils and high non-specific IgE levels. The MAST48 results showed no allergic sensitization in the three patients, whereas PKU-4 exhibited egg allergy. Table 6 shows the results for peripheral blood lymphocyte subsets, including regulatory T cells (Tregs). The results for CD19 + B cells, CD3 + T cells, CD3 + CD4 + T cells, and CD3 + CD8 + T cells were within the reference ranges for all four patients. Reference values for Tregs were based on the study by Luo et al. (19). PKU-1, −2, and −3 displayed a relatively high proportion of Tregs, whereas PKU-4 had a relatively low proportion of Tregs.

**Table 5:** Immunological profiles of patients with PKU.

PKU patient no.	1	2	3	4
Eosinophils (/μL)	131.6	74.3	134.5	167.4
Non-specific IgE (IU/mL)	<25	<25	<25	661
Serum-specific IgE	All Class 0	All Class 0	All Class 0	House dust: Class 3
				Mugwort: Class 1
				Japanese cedar: Class 6
				Hinoki: Class 3
				Elm Tree: Class 2
				Mites Mix: Class 3
				Ovomucoid: Class 2
				Egg white: Class 3

**Table 6:** Peripheral blood lymphocyte subsets of patients with PKU.

PKU patient no.		1	2	3	4
CD19^+^	B cells (%)	6.53	8.08	7.22	12.51
CD3^+^	T cells (%)	84.18	75.96	74.93	65.95
CD3^+^CD4^+^	Helper T cells (%)	42.14	57.6	66.76	41.7
CD3^+^CD8^+^	Cytotoxic T cells (%)	51.6	32.81	25.58	44.29
CD3^+^CD4^+^CD25^+^Foxp3^+^	Regulatory T cells (% median [range])*	3.12 (1.77 [1.15–2.83])	2.13 (0.81 [0.24–2.01])	2.29 (0.81 [0.24–2.01])	1.77 (0.81 [0.24–2.01])

*Reference values for Luo et al. (19).

### 3.4 Gut microbiota composition

**Figure 1:**
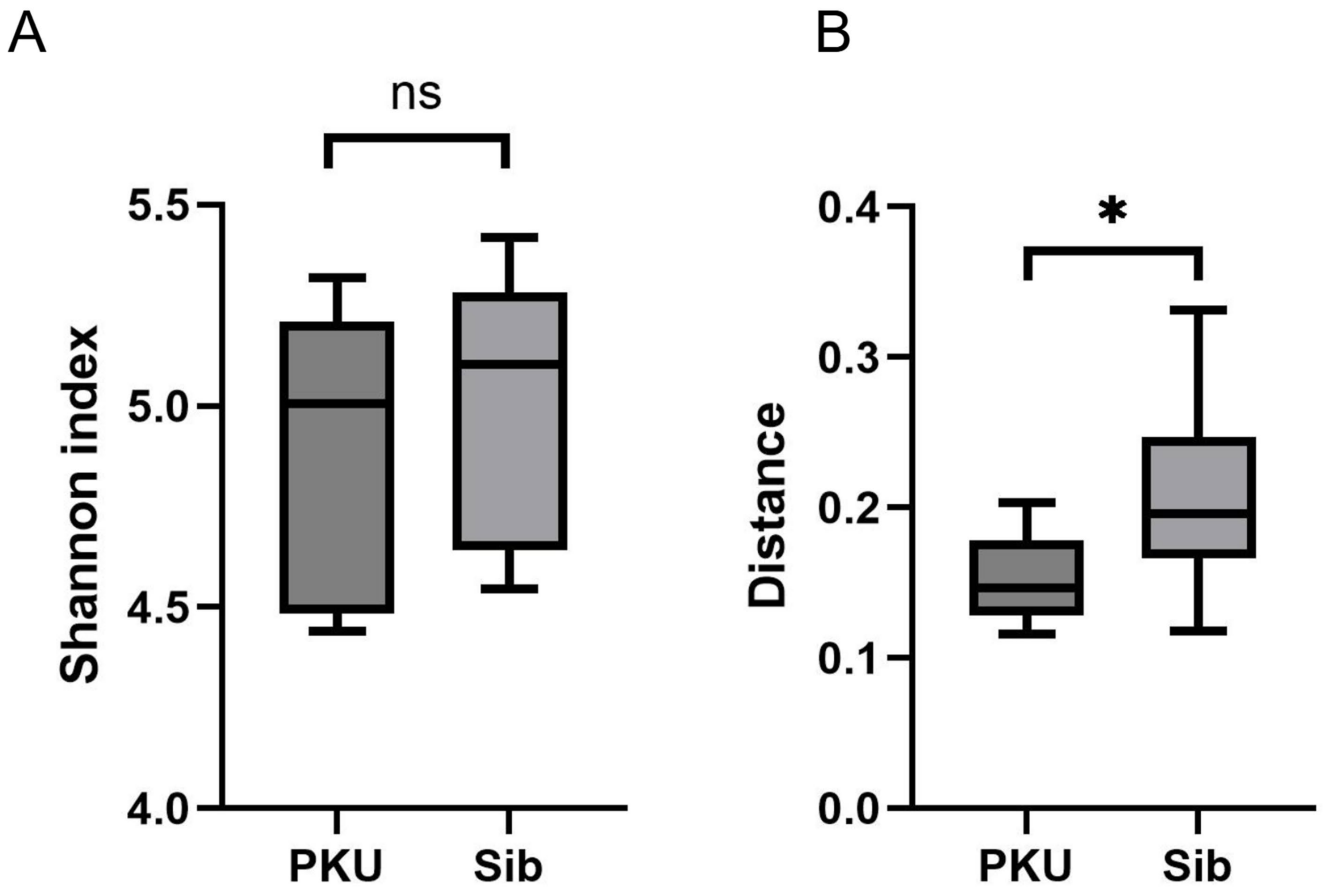
Diversity analysis of the gut microbiome. **(A)** Alpha diversity of the microbial communities in the PKU and Sib groups. Shannon index, *p* = 0.56. ns, not significant. **(B)** Beta diversity of the microbial communities in the PKU and Sib groups. Weighted UniFrac Distance, *p < 0.05.

Alpha diversity analysis revealed no significant differences between the PKU and Sib groups for any of the metrics used (first time: Chao1, *p* = 0.564; Shannon, p = 0.564; Simpson, *p* = 0.149; second time: Chao1, *p* = 0.825; Shannon, *p* = 0.827; Simpson, p = 0.827). According to the weighted UniFrac distances, beta diversity analysis showed that the structure of the PKU fecal microbiota differed significantly from that of the Sib group (*p* = 0.027) (Figure 1).

**Figure 2:**
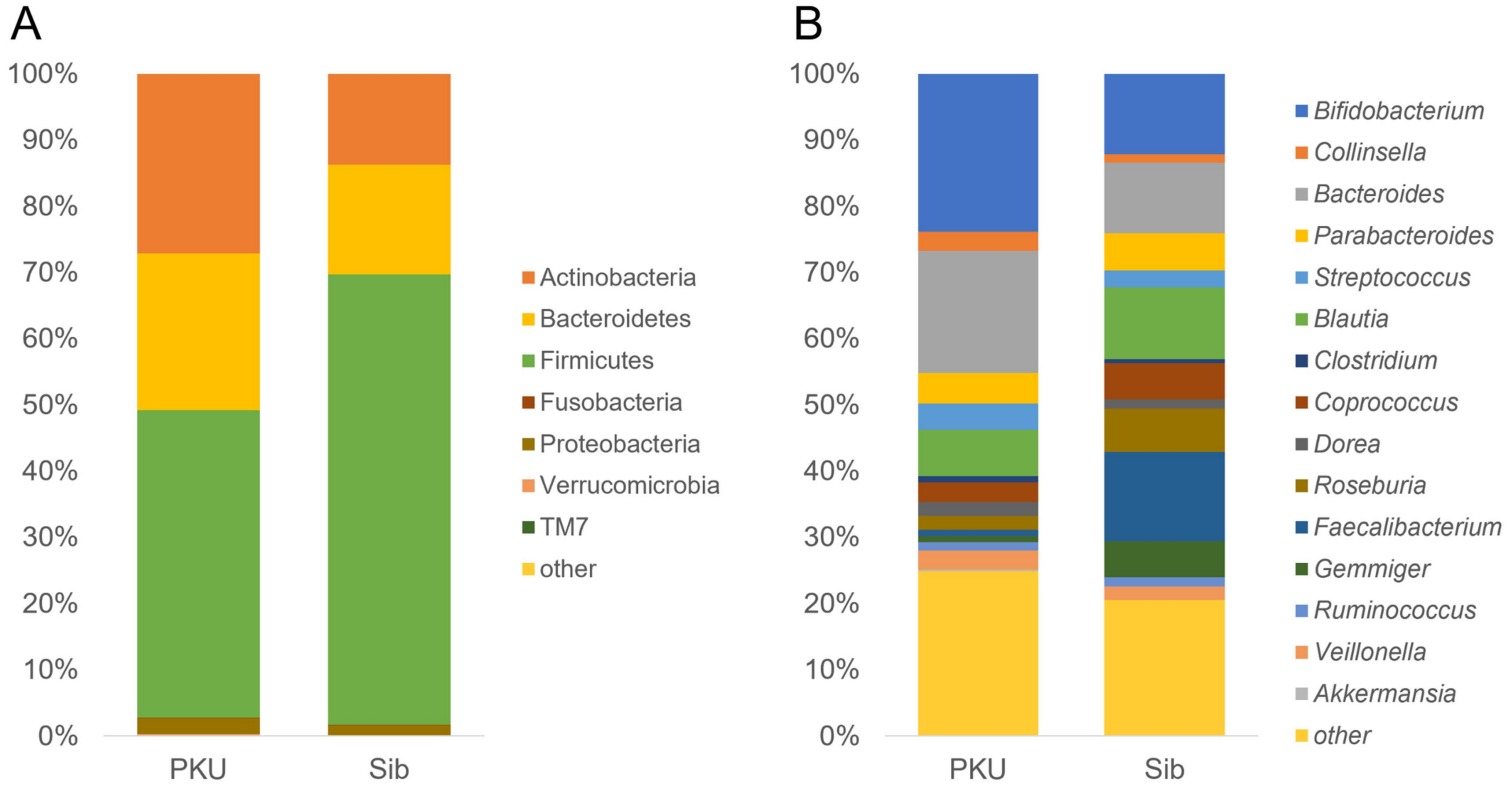
Gut microbiota composition in the PKU and Sib groups. Relative abundance of the gut microbiota at the **(A)** phylum and **(B)** genus level. The average values were calculated from sample results for *n* = 7, including two measurements from three families and one measurement from a single family.

**Figure 3:**
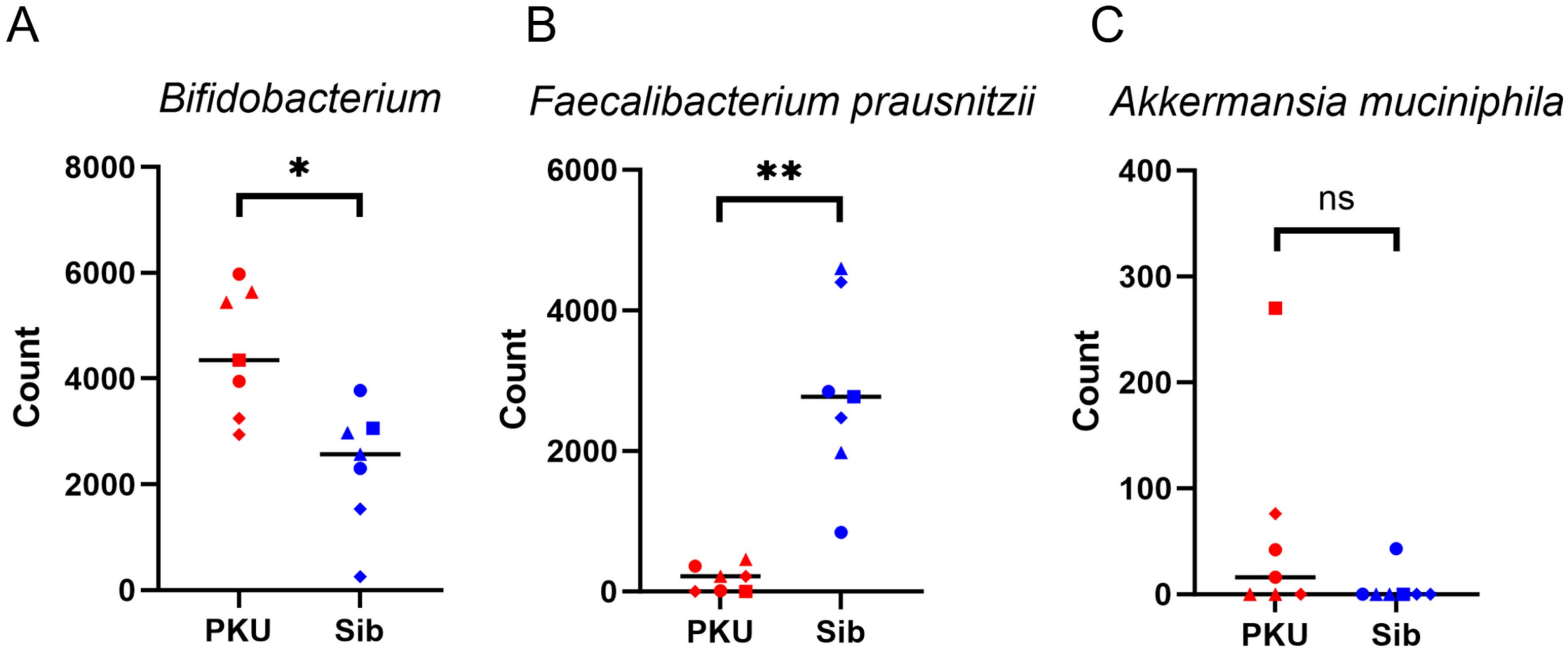
Comparison of the specific bacterial composition between the PKU and Sib groups. **(A)** Comparison of *Bifidobacterium* count data between the two groups. **(B)** Comparison of *Faecalibacterium prausnitzii* count data between the two groups. **(C)** Comparison of *Akkermansia muciniphila* count data between the two groups. Sample results for *n* = 7 were analyzed, including two measurements from three families and one measurement from a single family. The PKU group is represented in red, while the Sib group is shown in blue. Within each family, individual samples are denoted as follows: No. 1 (●), No. 2 (■), No. 3 (▲), and No. 4 (♦). ns, not significant; *p < 0.05, **p < 0.01 (Mann–Whitney’s U test using JMP software).

The results of the bacterial species identification of the gut microbiome at the phylum and genus levels are shown in Figure 2. At the phylum level, three groups, Firmicutes, Actinobacteria, and Bacteroidetes, accounted for more than 95% of the bacteria in both groups. The PKU group exhibited a decreased proportion of Firmicutes and an increased proportion of Actinobacteria compared with the Sib group. At the genus level, the PKU group showed a reduced proportion of *Faecalibacterium* and an elevated proportion of *Bifidobacterium* compared with the Sib group. At the species level, the PKU group showed a notably reduced proportion of *Faecalibacterium prausnitzii*, a major butyrate-producing bacterium (*p* = 0.0021). A statistically significant difference was observed in the compositions of two specific bacteria between the two groups (Figures 3A,B). Among *Bifidobacterium*, the lowest abundances were recorded for PKU-4 and Sib-4 within their respective groups. The observed shifts in *Faecalibacterium* and *Bifidobacterium* abundance were consistently reproducible across iterative analyses. The abundance of other butyrate-producing bacteria, such as *Anaerostrips*, *Roseburia*, and *Coprococcus*, did not differ significantly between the two groups. In contrast, *Akkermansia muciniphila*, a mucin-degrading bacterium, was more abundant in the PKU group than in the Sib group (Figure 3C).

### 3.5 SCFA concentrations in the fecal samples

There was no significant difference in the total SCFA levels between the PKU and Sib groups (Table 7). However, butyrate levels were slightly lower and acetate and propionate levels were slightly higher in the PKU group than those in the Sib group, resulting in a significant elevation in the acetate/butyrate and propionate/butyrate ratios. These ratios showed clear differences between groups, with bootstrap *p*-values < 0.001, and 95% confidence intervals that did not cross zero (0.91–5.77 and 1.02–2.07, respectively). This decrease in butyrate production was particularly evident in patient PKU-4.

**Table 7:** SCFA concentrations in the fecal samples.

Subject No.	PKU-1	PKU-3	PKU-4	Sib-1	Sib-3	Sib-4	Median difference (PKU-Sib)	95% CI (percentile)	Bootstrap *p*-value
Total (mg/g)	2.95	5.58	2.23	4.27	2.54	2.74	0.21	−2.04−3.04	0.65
Acetate (mg/g)	1.83	2.42	1.49	1.86	1.18	1.59	0.24	−0.37−1.24	0.70
Propionate (mg/g)	0.8	1.72	0.53	0.63	0.78	0.37	0.17	−0.25−1.35	0.58
iso-Butyric acid (mg/g)	–	0.14	–	0.11	–	–			
Butyrate (mg/g)	0.32	0.73	0.21	1.4	0.58	0.66	−0.34	−1.19−0.15	0.38
iso-Valeric acid (mg/g)	–	0.29	–	0.12	–	0.12			
Valeric acid (mg/g)	–	0.28	–	0.15	–	–			
Acetate/Butyrate	5.72	3.32	7.10	1.33	2.03	2.41	3.69	0.91–5.77	<0.001
Propionate/Butyrate	2.5	2.36	2.52	0.45	1.34	0.56	1.94	1.02–2.07	<0.001

## 4 Discussion

It is well known that diet changes the gut microbial ecology and affects host physiology. This pilot study explored how dietary therapy for PKU influences gut microbiota and considered whether these changes might be related to food allergy incidence. Our results showed that the dietary therapy for PKU leads to significant alterations in gut microbiota composition. Specifically, children with PKU exhibited a reduced abundance of *Faecalibacterium prausnitzii* compared to their unaffected siblings, while *Bifidobacterium* was more abundant. However, no overall decrease in total SCFA levels was observed in the PKU group, and a modest increase in peripheral blood Tregs was noted. This study provides the first detailed comparison of gut microbiota in children with PKU and their unaffected siblings, raising the hypothesis that the characteristic microbial profile in PKU may contribute to their unique immunological background.

A survey conducted during this research revealed a notably low prevalence of physician-reported food allergies among children with PKU, at approximately 1%. By contrast, Japanese birth cohort studies that enrolled 2,000–4,000 participants per age stratum have reported food allergy prevalence of 5–10% in infants (20). Although cross-study comparisons warrant caution, this discrepancy prompted us to consider whether diet-associated shifts in the gut microbiota might contribute to the observed difference. As part of PKU management, the recommended diet should be started early after diagnosis to prevent various symptoms, including neurological disorders (2). Infancy and early childhood are critical periods for the acquisition and maturation of gut microbiota. During this period, environmental factors can have a significant impact on the gut microbiota. Several studies have investigated alterations in gut microbiota among children with PKU, including comparisons with healthy controls (8) and those with mild hyperphenylalaninemia (9). As genetic and environmental factors, including diet, drugs, and prebiotics, are associated with microbiota diversity and composition (21–24), we consider that comparing the gut microbiota of children with PKU with their unaffected siblings would help reduce bias related to maternal and environmental factors. As a result, this study, while limited by a small sample size, focused on comparing the gut microbiota and SCFA of four children with PKU and their unaffected siblings.

Alpha diversity tended to be slightly lower in the PKU group, although the difference was not statistically significant. In contrast, beta diversity differed significantly between the two groups, suggesting that the PKU and Sib groups exhibited different microflora compositions. Specifically, the PKU group exhibited a decreased proportion of *Faecalibacterium* and an elevated proportion of *Bifidobacterium* compared with the Sib group. The PKU group had a notably reduced proportion of *F. prausnitzii*, one of the most important butyrate-producing bacteria, consistent with previous reports (9). Dietary intervention for children with PKU is high in carbohydrates. Staple low-protein rice is rich in simple carbohydrates, from which proteins are removed via enzymatic processing. It has been suggested that fast-absorbing carbohydrates are not suitable substrates for *F. prausnitzii* growth (9, 25, 26) and may be the cause of its depletion. *F. prausnitzii* is also a biomarker for inflammatory bowel disease (27); however, fecal calprotectin concentrations are not elevated in patients with PKU, and no evidence of intestinal inflammation has been found in these patients (9, 28). Interestingly, we observed that the proportion of *Bifidobacterium* was higher in children with PKU than in their siblings. The genus *Bifidobacterium* is one of the main groups of intestinal microflora in the large intestine that uses sugar to produce lactic acid and acetic acid. *Bifidobacterium* plays a key role in disease prevention, including intestinal mucosal immune development, host defense against infections, and protection against allergy and obesity (29–33). An increased proportion of *Bifidobacterium* has been reported in studies on adult PKU (10) but not previously in children (8, 9). Our study showed that the proportion of *Bifidobacterium* increased from childhood, indicating that dietary interventions in the neonatal period did not inhibit *Bifidobacterium* colonization. The stable colonization of *Bifidobacterium* is associated with immune tolerance in the intestinal tract by increasing the number of Tregs and inducing apoptosis of mast cells (34, 35). In early infancy, which is prone to immune overinduction, the predominance of *Bifidobacterium* in the gut microbiota, which has immunoprotective properties, may be associated with preventing the later development of allergic diseases (36). Additionally, *A. muciniphila* was detected more frequently in the PKU group than in siblings. This mucin-degrading bacterium predominantly produces acetate and propionate and has been implicated in maintaining epithelial barrier integrity and modulating inflammatory responses (37). Our observation was consistent with previous studies detecting *Akkermansia* in the PKU population (8, 10). Preclinical mouse study suggested its immunomodulatory and protective roles, although these findings are not directly translatable to humans (38). Taken together, the relative enrichment of *Bifidobacterium* species and the higher detection frequency of *A. muciniphila* represent intriguing microbiota changes, leading to the hypothesis that the distinctive intestinal microenvironment in PKU may be inversely associated with the development of food allergy.

No remarkable decrease in total SCFA levels was observed in the PKU group in our dataset, while butyrate concentration was relatively low. SCFAs, mainly acetate, propionate, and butyrate, have profound effects on gut health as they are energy sources, inflammation modulators, and a part of gut motility and wound healing. Specifically, SCFAs are energy substrates for the colonic epithelium and peripheral tissues (7, 39, 40). Verduci et al. also reported a decrease in total SCFA levels, particularly butyrate, in the gut microbiota of patients with PKU (41). Although the reduction in butyrate concentration was not remarkable in this investigation, the acetate/butyrate ratio and propionate/butyrate ratio were significantly higher in the PKU group than in the Sib group. The increase in acetate levels was attributed to the high proportion of *Bifidobacterium*, while the increase in propionate levels might be due to a relatively high proportion of *Veillonella*, a propionate-producing bacterium, as observed in the PKU group. However, further validation is needed to clarify the impact of these byproducts on host homeostasis.

Tregs are specialized for immunosuppressive functions and are also important in controlling allergic diseases (42). Tregs are essential for establishing oral tolerance, and those present in the gut are mainly induced by gut microbiota and food antigens. The proportion of CD4 + CD25 + Tregs in the peripheral blood of patients with food allergies was lower than that in healthy individuals or those who are cured of food allergies (43). A reduced butyrate level in the gut is likely to be involved in the development of allergies, as it affects immune regulation by Tregs (44). Patients with food allergies and atopic dermatitis have fewer butyrate-producing bacteria than healthy individuals (45). However, in the present study, no reduction in the number of Tregs was observed in the PKU group. This suggests that the effect of the decrease in butyrate due to the decrease in the proportion of *F. prausnitzii* is limited. In contrast, *Bifidobacterium* has been suggested to contribute to the development of Tregs in early infancy, when the thymus is still underdeveloped (46). It has also been reported that the proportion of Foxp3 + Tregs in the spleen increases after a single dose of *Bifidobacterium* in mice, ameliorating inflammation in allergic asthma and elevating IgE levels in food allergy (47). Therefore, a possible mechanism may be that the increase in *Bifidobacterium* plays some protective role in avoiding a decrease in the proportion of Tregs or the onset of allergies. In this study, only one patient (PKU-4), who had an allergic background, tended to have lower butyrate levels and fewer Tregs than the other children with PKU. In addition, the proportion of *Bifidobacterium* in PKU-4 was lower than in the other children with PKU included in this study. Family background may also be involved, as Sib-4 had a lower proportion of *Bifidobacterium* than the other individuals in the Sib group.

Recent hypotheses on the mechanism of food allergy development suggest that sensitization to allergens occurs through environmental exposure via the skin (3–5). Since the Phe-free formula is not derived from natural food sources, it does not act as an allergen in the development of food allergies. Children with PKU, like healthy children, are exposed to environmental food allergens. However, based on our observations, children with PKU who adhere to appropriate dietary therapy tend to have fewer instances of eczema, potentially reducing the likelihood of percutaneous sensitization. This aligns with our finding that the prevalence of food allergies is lower in children with PKU. Notably, food allergy was observed in only one child, PKU-4, who had a history of eczema from infancy, suggesting a possible higher susceptibility to percutaneous sensitization.

In parallel with the gut microbiota and immunological evaluation, the diet therapy needs further improvement. Individuals with PKU need to consume a low Phe diet throughout their lives, and the missing energy and protein should be supplemented with artificial amino acid mixtures. Glycomacropeptide (GMP) is a protein derived from cheese whey and contains a minimal amount of Phe (48, 49). Recently, companies involved in producing PKU therapeutic diets have been working to produce specific medical foods based on GMP. Mouse models suggest a prebiotic effect on beneficial gut bacteria (50). A study that compared the gut microbiota before and after 6 months of GMP supplementation in nine patients with PKU showed an increased percentage of the butyrate-producing bacterium *Agatobacter*, suggesting a beneficial effect of GMP intake (50, 51). Using natural food-based GMP products may be one beneficial way to improve the dietary management of PKU.

The most evident limitation of this study is the small cohort size, consisting of only four children with PKU and their healthy siblings. However, given the rarity of PKU in Japan and the limited opportunity to include unaffected siblings, this was unavoidable. Another limitation is the absence of questionnaire data from age-matched controls, as well as the lack of detailed dietary records and immunological information from the unaffected sibling controls. In addition, the gut microbiota of children with PKU was assessed only after initiation of dietary therapy, making it unclear whether the observed changes reflect the underlying disease or the effects of dietary intervention. However, based on observations in adults showing microbiota shifts following dietary liberalization (11), these alterations are likely to reflect the effects of dietary intervention. With respect to food allergy, oral food challenges were not performed; therefore, clinical food allergy could not be definitively confirmed. It is conceivable that some children with PKU may never have consumed certain allergenic foods; however, PKU dietary management restricts rather than eliminates natural protein, and such foods are therefore not necessarily absent from the diet. The amount of oral intake may have remained below individual reaction thresholds, potentially underestimating the prevalence of food allergy in this cohort, which we acknowledge as a limitation. Nevertheless, it is noteworthy that food allergies have not been reported in adults with reduced adherence to dietary therapy or in those whose diets have been liberalized through treatment (6, 11), and this observation is consistent with our own clinical experience. Finally, this study alone could not elucidate the mechanisms by which changes in specific bacterial species may influence host immunity and suppress the development of allergy.

In conclusion, dietary therapy significantly altered the gut microbiota of children with PKU compared with their unaffected siblings. However, no clear evidence of dysbiosis or adverse effects on immune function was observed in the PKU group. Given the lower frequency of physician-reported food allergy in the PKU group, we advance a working hypothesis: in diet-adherent PKU, host intestinal homeostasis may be maintained through a distinctive microbial balance, in which the reduction of butyrate-producing bacteria is compensated by acetate-producing bacteria, and this balance could be negatively associated with the development of food allergy. Further studies in larger PKU cohorts over extended periods would be valuable in refining dietary strategies and improving the nutritional quality of PKU management. In addition, integration of host metabolomic and transcriptomic analyses would help to clarify the causal relationship between gut microbiota changes and the low prevalence of food allergies in this population.

## Data Availability

Due to Institutional Review Board and informed consent restrictions in this rare-disease cohort, access to the raw data is limited to protect participant confidentiality, and access will be permitted upon reasonable request by qualified researchers, subject to institutional approval and completion of a data use agreement.
